# Predictors of long‐term weight loss trajectories during a behavioral weight loss intervention: An exploratory analysis

**DOI:** 10.1002/osp4.530

**Published:** 2021-05-19

**Authors:** Danielle M. Ostendorf, Jennifer M. Blankenship, Laura Grau, Jaron Arbet, Nia S. Mitchell, Seth A. Creasy, Ann E. Caldwell, Edward L. Melanson, Suzanne Phelan, Daniel H. Bessesen, Victoria A. Catenacci

**Affiliations:** ^1^ Department of Medicine Anschutz Health and Wellness Center University of Colorado Anschutz Medical Campus Aurora Colorado USA; ^2^ Department of Medicine Division of Endocrinology, Metabolism, and Diabetes University of Colorado Anschutz Medical Campus Aurora Colorado USA; ^3^ Department of Biostatistics and Informatics Colorado School of Public Health University of Colorado Anschutz Medical Campus Aurora Colorado USA; ^4^ Department of Medicine Duke University School of Medicine Durham North Carolina USA; ^5^ Department of Medicine Division of Geriatric Medicine University of Colorado Anschutz Medical Campus Aurora Colorado USA; ^6^ Eastern Colorado Veterans Affairs Geriatric Research, Education, and Clinical Center Denver Colorado USA; ^7^ Department of Kinesiology & Public Health California Polytechnic State University San Luis Obispo California USA

**Keywords:** lifestyle modifications, obesity phenotypes, obesity treatment, psychosocial variables, race

## Abstract

**Background:**

Substantial interindividual variability in response to behavioral weight loss interventions remains a critical challenge in obesity treatment. An improved understanding of the complex factors that contribute to this variability may improve obesity treatment outcomes.

**Objective:**

To identify weight change trajectories during a behavioral weight loss intervention and to explore differences between trajectory groups in sociodemographic, biologic, behavioral, and psychosocial factors.

**Methods:**

Adults (*n* = 170, 40 ± 9 years, BMI 34 ± 4 kg/m^2^, 84% female) participated in an 18‐month behavioral weight loss intervention. Weight was measured at 0, 3, 6, 9, 12, 15, 18, and 24 months. Among participants with at least two weights after baseline (*n* = 140), clusters of longitudinal trajectories of changes in weight were identified using a latent class growth mixture model. The association between baseline factors or changes in factors over time and trajectory group was examined.

**Results:**

Two weight change trajectories were identified: “weight regainers” (*n* = 91) and “weight loss maintainers” (*n* = 49). Black participants (90%, 19/21) were more likely than non‐Black participants to be regainers versus maintainers (*p* < 0.01). Maintainers demonstrated greater increases in device‐measured physical activity, autonomous motivation for exercise, diet self‐efficacy, cognitive restraint, and engagement in weight management behaviors and greater reductions in barriers for exercise, disinhibition, and depressive symptoms over 24 months versus regainers (*p* < 0.05).

**Conclusion:**

Maintainers and regainers appear to be distinct trajectories that are associated with specific sociodemographic, behavioral, and psychosocial factors. Study results suggest potential targets for more tailored, multifaceted interventions to improve obesity treatment outcomes.

**Abbreviations**
ADOPT: accumulating data to optimally predict obesity treatment
BMI: body mass index
CES‐D: Center for Epidemiologic Studies Depression Scale
FDR: false discovery rate
METs: metabolic equivalents
MVPA: moderate‐to‐vigorous physical activity
PA: physical activity
SD: standard deviation
SE: standard error
TFEI: Three‐Factor Eating Inventory
TSRQ: Treatment Self‐Regulation Questionnaire

## 1 INTRODUCTION

Increasing the proportion of people who are able to lose weight and maintain their weight loss long‐term is perhaps the most significant challenge in obesity treatment. During weight loss, energy expenditure declines, appetite increases, and metabolism shifts in a manner that favors energy intake and storage.^1^, ^2^ These changes in the key factors regulating body weight produce a strong biological drive to regain lost weight. The behavioral and cognitive work required to lose and sustain weight loss is not reduced over time and the rewards (progressive weight loss) wane.^3^ These biologic, behavioral, and psychosocial factors likely contribute to the substantial interindividual variability in response to a weight loss intervention.^4^ A clearer understanding of the factors that explain this heterogeneity in weight loss can lead to the development of precision‐medicine approaches that will have greater long‐term effectiveness.^3^

Traditional analytic approaches to understand treatment response in obesity research focus on the mean weight loss response of a sample of participants or the percent of participants achieving a predefined threshold of clinically meaningful weight loss (commonly ≥5%–10% weight loss). While there is value in these approaches, the dynamic changes in weight over time and the substantial interindividual variability observed in treatment responses are masked.^4^ Recently, data‐driven approaches, such as latent class analysis and growth mixture modeling, have been applied to weight loss interventions to improve our understanding of the interindividual variability in weight loss response.^5^, ^6^, ^7^, ^8^, ^9^ These approaches utilize longitudinal data to identify groups of individuals who demonstrate similar patterns of weight change over time. Previous studies have identified different weight loss trajectories during short‐term (3–4 months) and longer‐term (12–24 months) lifestyle weight loss interventions^5^, ^6^, ^7^, ^8^, ^10^, ^11^ or following bariatric surgery for up to 7 years.^9^ Some studies have extended these analyses to determine factors associated with weight change trajectories.^5^, ^6^, ^7^, ^9^, ^10^, ^11^ However, these factors have been limited to baseline sociodemographic factors or a few diet and behavioral variables. These studies lay the foundation for using data‐driven approaches to understand the individual variability in response to weight loss interventions. However, few studies have examined a comprehensive array of sociodemographic, biologic, behavioral, and psychosocial factors that potentially predict different weight change trajectories. Exploratory analyses are a critical first step towards developing tailored interventions to promote long‐term weight loss maintenance.^3^

Data from a recently completed 18‐month behavioral weight loss intervention^12^ provided a unique opportunity to perform an in‐depth exploration of factors that may relate to different weight change trajectories. These exploratory analyses allow identification of subpopulations which may respond more or less favorably to behavioral weight loss interventions and ultimately may provide insight into targets to improve future interventions. Therefore, the purpose of this secondary data analysis was to (1) identify groups of individuals with distinct patterns of weight change over 24 months in response to a behavioral weight loss intervention and (2) explore differences between groups in sociodemographic, biologic, behavioral, and psychosocial factors. This analysis was exploratory in nature, so there were no *a‐priori* hypotheses.

## 2 METHODS

### 2.1 Participants

**FIGURE 1:**
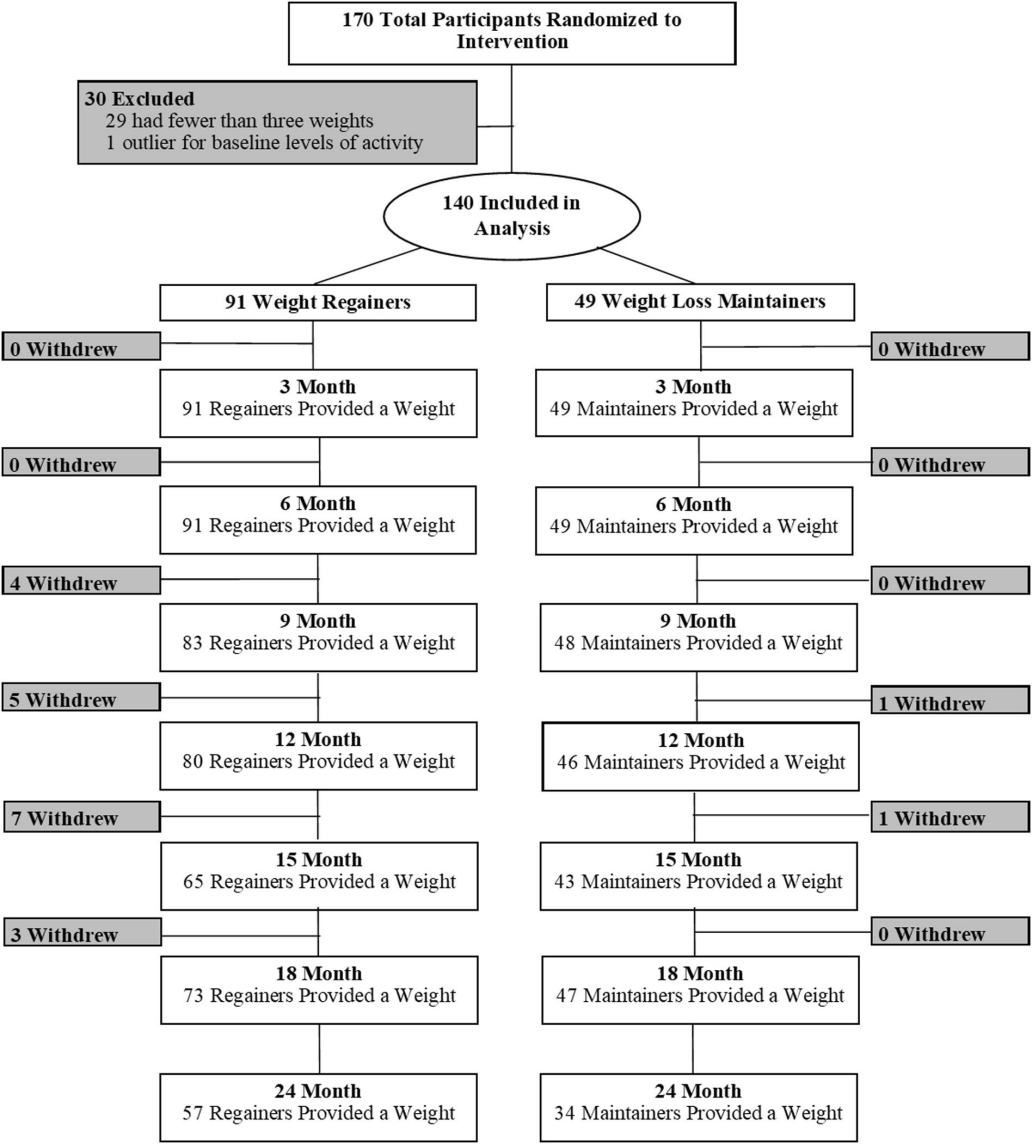
Consort Diagram

A secondary analysis was conducted and included 140 adults with overweight or obesity (81% female, 15% Black, BMI 34 ± 4 kg/m^2^) who participated a randomized controlled weight loss trial which included an 18‐month comprehensive behavioral weight loss intervention and follow‐up measures at 24 months (NCT01985568).^12^ The weight loss trial was designed to determine the optimal time to initiate exercise during a behavioral weight loss intervention. A detailed description of the methods as well as the primary results of the trial have been published previously.^12^ The trial was approved by the Colorado Multiple Institutional Review Board. Participants included adults (18–55 years) with overweight or obesity (BMI 27–42 kg/m^2^) who lived or worked within 20 miles of University of Colorado Anschutz Health and Wellness Center. Exclusionary criteria have been previously described in detail.^12^ Participants were excluded if they had >5% weight loss over the past 6 months and self‐reported performing >150 min/week of at least moderate intensity exercise, based on the first Edition Physical Activity (PA) Guidelines for Americans for overall health.^13^ For this secondary data analysis, participants were included if they had at least three weight measures, including baseline weight (Figure 1).

### 2.2 Interventions

Participants in the weight loss trial were randomized 1:1 to one of two groups: standard behavioral therapy or sequential behavioral therapy, stratified by sex. Both randomized groups received an identical 18‐month group‐based behavioral weight loss program and 6‐month supervised exercise program. The only difference between randomized groups was the timing of exercise initiation. Those in the standard group received the supervised exercise program during months 0–6 whereas those in the sequential group received the supervised exercise program during months 7–12. All participants received group‐based comprehensive behavioral weight loss support, led by a registered dietician nutritionist. Additional details of the behavioral weight loss program have been described previously.^12^

### 2.3 Sociodemographic factors

Age, gender, race, ethnicity, and education were self‐reported at baseline.

### 2.4 Biologic factors

Body weight was measured with a calibrated digital scale (to the nearest 0.1 kg; Tanita® PH‐740) and waist circumference (in centimeters) was measured at the level of the superior iliac crest at months 0, 3, 6, 9, 12, 15, 18, and at a 24‐month follow‐up. Body composition (percent fat mass) was measured using the dual‐energy x‐ray absorptiometry (Hologic Discovery QDR series; Hologic) at months 0, 6, 12, and 18. Cardiorespiratory fitness was measured with a maximal aerobic capacity test (VO_2_max) at months 0, 6, 12, and 18 using a modified Balke treadmill protocol^14^ and indirect calorimetry (Parvo Medic Truemax 2400).

### 2.5 Behavioral factors

All other predictors were assessed at months 0, 6, 12, 18, and 24, unless otherwise stated. Free living moderate‐to‐vigorous PA (MVPA) was measured with the SenseWear Mini Armband (version 7.0; BodyMedia Inc.). At each time point, participants were asked to wear the SenseWear Mini for 24 h/day over seven consecutive days. To be included in analyses examining PA, ≥4 valid days, including ≥1 weekend day were required. A day was classified as valid if the device was worn ≥22.8 h/day (95% wear time requirements).^12^ A customized program was used to calculate “bout MVPA” as the sum of minutes of moderate‐to‐vigorous intensity activity accumulated in bouts lasting ≥10 min in duration where >80% of the entire bout was spent in ≥3 METs.^15^

Energy intake and diet macronutrient content was measured using 3‐day diet records at months 0, 6, 12, and 18. Diet records were analyzed using Nutrition Data System for Research software (version 2016; Nutrition Coordinating Center, University of Minnesota) by personnel blinded to randomized group assignment.

### 2.6 Psychosocial factors

Self‐efficacy for exercise was assessed with the Barriers Self‐Efficacy Scale^16^; scores range from 0–10, with higher scores indicating greater self‐efficacy. Perceived benefits and barriers of exercise were assessed with the Exercise Benefits and Barriers Scale^17^ and separate scores were provided for benefits (range 29–116) versus barriers (range 14–56); higher scores indicate greater perceived benefits/barriers. Exercise motivation was assessed using the Behavioral Regulations for Exercise Questionnaire^18^, which provides separate scores (range 0–4) for external regulation (“I exercise because others say I should”), introjected regulation (“I feel guilty when I don't exercise”), identified regulation (“I value the benefits of exercise”), and intrinsic regulation (“I exercise because it is fun”). Higher scores indicate greater levels of motivation.

Eating self‐efficacy was measured with the Weight Exercise Lifestyle questionnaire,^19^ using the total score (range 0–189), with higher scores indicating greater self‐efficacy. Cognitive restraint (i.e., tendency to consciously restrict food intake, range 0–21), hunger (range 0–14), and disinhibition (i.e., tendency to overeat in the presence of palatable foods, range 0–16) were assessed using the Three‐Factor Eating Inventory (TFEI)^20^.

Weight management behaviors were assessed with the Eating Behavior Inventory (Revised^21^), using the total score (sum of all items divided by 30, range 1–4); higher scores indicate greater engagement in weight management behaviors. Motivation for engaging in weight loss treatment was assessed using the Treatment Self‐Regulation Questionnaire (TSRQ,^22^), which provides scores for autonomous or controlled motivation for treatment (range 0–7); higher scores indicate greater motivation. At baseline, the TSRQ for Entering Treatment was used, and at all remaining time points, the TSRQ Concerning Continued Program Participation was used. Depressive symptoms were measured using the Center for Epidemiologic Studies Depression Scale (range 0–60); higher scores indicate greater depressive symptoms, and scores ≥16 identify individuals at risk for clinical depression.^23^

### 2.7 Statistical analysis

All participants provided a baseline weight. However, participants (*n* = 29) were excluded from this secondary data analysis if they had fewer than two outcome weight measures after starting the intervention. Any weights that occurred after a participant withdrew from the intent‐to‐treat trial were excluded from the analysis. One participant was excluded because their device‐measured total MVPA level (954 min/week) at baseline was considered an extreme outlier, and outside of the exclusion criteria for the primary study. Thus, 140 participants were included in this analysis (Figure 1).

A latent class growth mixture model (lcmm R package^24^) was used to identify distinct clusters of longitudinal weight change trajectories over 24 months in response to the behavioral weight loss intervention. Given that change in weight at 18 months was not different by randomized group,^12^ randomized groups were combined to increase sample size. However, randomized group was tested as a covariate in analyses. Covariates were not included during the mixture modeling process (i.e., when determining the optimal number of clusters).^25^ Models were estimated iteratively by increasing the number of clusters until the best‐fitting model was found.^26^ Clusters with the lowest Bayesian Information Criteria were selected. Clusters with a sample size <10 were not considered (Table S1).

Chi‐Squared tests or fisher's exact tests for categorical variables and two sample *t*‐tests for continuous variables were used to explore associations between trajectory group and sociodemographic, biologic, behavioral, and psychosocial factors measured at baseline. To explore whether change in different continuous variables was associated with trajectory group, generalized estimating equations were used, with each continuous variable as the outcome and month, trajectory group, and a group*month interaction as covariates. Note, the test of the group*month interaction assessed whether change in a variable over time differed between weight loss trajectory groups. To examine the association between adherence to the dietary and PA prescriptions, logistic generalized estimating equations were used with a binary longitudinal variable as the outcome and month, trajectory group, and a group*month interaction as covariates. A participant was defined as adherent to the dietary prescription if their estimated energy intake was at or below their prescribed calorie goal at each time point. A participant was defined as adherent to the PA prescription if their device‐measured bout MVPA was at or above 300 min/week at each time point (months 6, 12, and 18 for standard; months 12, 18, and 24 for sequential). Given the exploratory nature of this study, all results with *p* < 0.05 were considered statistically significant, and false discovery rate (FDR_adj_) multiple testing adjusted *p*‐values were also reported.^27^ No *a‐priori* power analysis was conducted.

## 3 RESULTS

### 3.1 Weight loss trajectories

**FIGURE 2:**
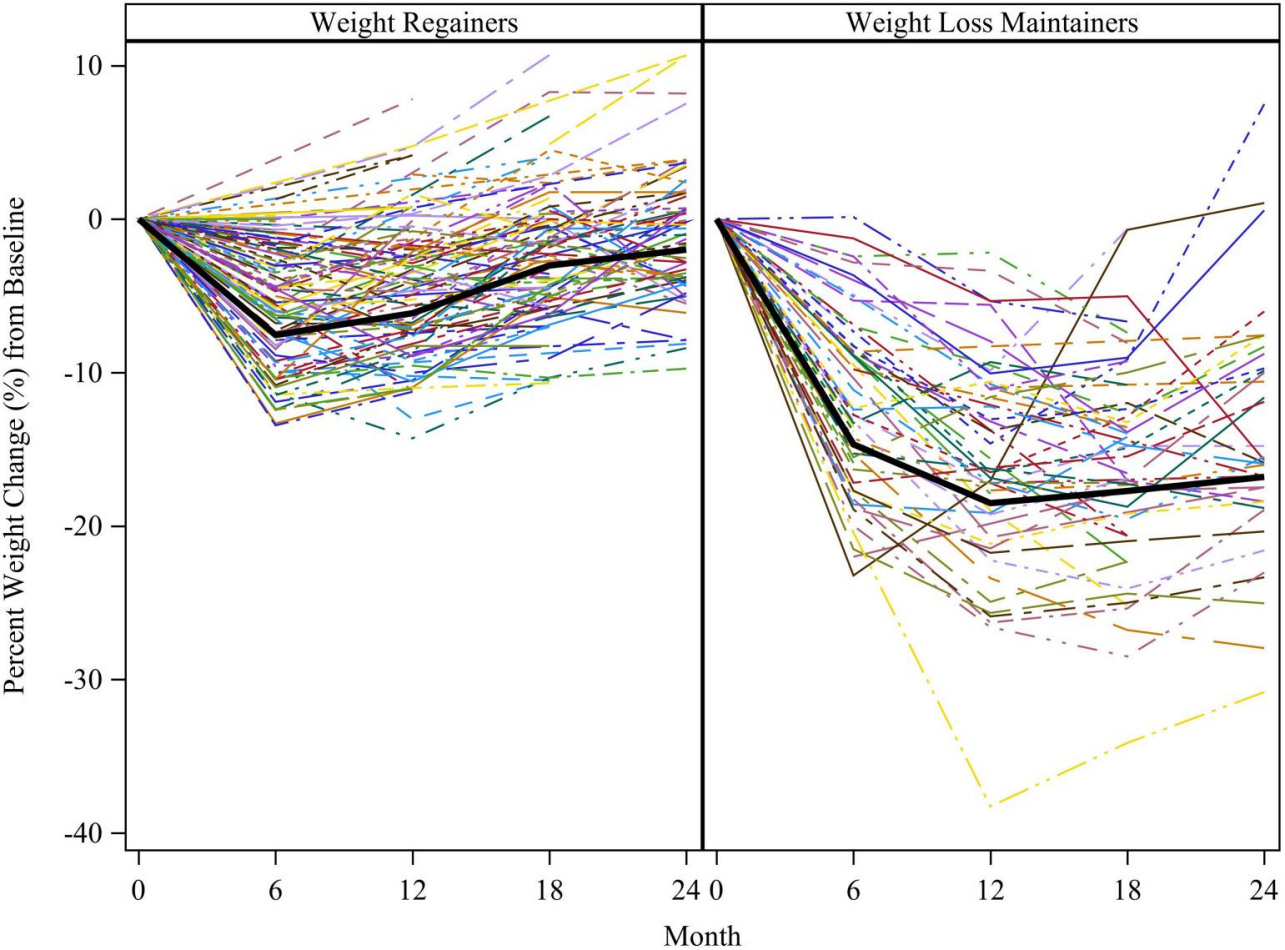
Weight loss trajectories. Weight loss trajectories represent a two‐cluster, cubic model, determined from latent class growth mixture model. Each color represents an individual participant in the study. Within the weight regainer group, average weight loss (95% CI) was 4.8% (4.2%, 5.4%) at 3 months, 5.9% (5.1%, 6.7%) at 6 months, 5.5% (4.6%, 6.5%) at 9 months, 4.7% (3.7%, 5.7%) at 12 months, 3.3% (2.2%, 4.4%) at 15 months, 2.3% (1.2%, 3.3%) at 18 months, and 1.2% (0.1%, 2.3%) at 24 months. Within the weight loss maintainer group, average weight loss (95% CI) was 8.4% (7.2%, 9.6%) at 3 months, 12.2% (10.5%, 14.0%) at 6 months, 14.6% (12.6%, 16.7%) at 9 months, 15.9% (13.8%, 18.1%) at 12 months, 16.7% (14.6%, 18.8%) at 15 months, 15.9% (13.9%, 18.0%) at 18 months, and 14.0% (11.1%, 16.8%) at 24 months.

A two‐cluster cubic model was selected as the best‐fitting model based on Bayesian Information Criteria (Table S1). The majority of participants (79%) had ≥6 weight measurements. The largest class, “weight regainers” (*n* = 91, 65%) was characterized by moderate weight loss at 3 months (4.8%, 95% CI: 4.2%–5.4%), followed by a steady regain back to baseline weight at 24 months, with a 24‐month average weight loss of 1.2% (95% CI: 0.1%–2.3%, Figure 2). The second class, “weight loss maintainers” (*n* = 49, 35%) was characterized by greater weight loss at 3 months (8.4%, 95% CI: 7.2%–9.6%) followed by continuous weight loss through 12 months (15.9%, 95% CI: 13.8%–18.1%), a plateau in weight from 12–18 months, and modest weight regain from 18–24 months, with a 24‐month average weight loss of 14.0% (95% CI: 11.1%–16.8%) (Figure 2). Weight loss at 24 months was significantly greater in maintainers compared to regainers (*p* < 0.01). Figure 2 demonstrates that while this analytical approach identified two distinct patterns of weight change, there was substantial individual variability in weight change within trajectory groups.

### 3.2 Baseline factors associated with trajectory group

Trajectory group was not associated with randomized intervention group or baseline age, gender, ethnicity, education, body weight, BMI, waist circumference, body fat (%), cardiorespiratory fitness (Table 1), or baseline behavioral factors (bout MVPA, energy and macronutrient intake, Table 2). Black participants (19/21, 90%) were more likely than non‐Black participants (72/119, 60.5%) to be classified as weight regainers (*p* = *0.01, FDR*_*adj*_ *p > 0.05*). Of the Black participants in the regainer group, 15 (79%) were women. Maintainers demonstrated higher baseline levels of exercise barriers (*p* = *0.01*, *FDR*_*adj*_ *p > 0.05*), lower intrinsic regulation for exercise (*p* < *0.01*, *FDR*_*adj*_ *p > 0.05*), lower eating self‐efficacy (*p* = *0.02, FDR*_*adj*_ *p > 0.05*), higher hunger *(p* = *0.02, FDR*_*adj*_ *p > 0.05*), and higher levels of depressive symptoms (*p* = *0.046*, *FDR*_*adj*_ *p > 0.05*) versus regainers (Table 2).

**TABLE 1:** Baseline sociodemographic and biologic predictors of trajectory membership

Baseline predictors	Total sample (*n* = 140)	Weight regainers (*n* = 91)	Weight loss maintainers (*n* = 49)	*p* value	FDR_adj_ *p* value
Randomized group				0.38	0.65
Standard	67 (48%)	46 (51%)	21 (43%)		
Sequential	73 (52%)	45 (49%)	28 (57%)		
Age (y)	40 ± 9	40 ± 9	40 ± 9	0.86	0.91
Gender				0.62	0.83
Women	114 (81%)	73 (80%)	41 (84%)		
Men	26 (19%)	18 (20%)	8 (16%)		
Race				**0.01** ^a^	0.11^a^
White	108 (77%)	67 (74%)	41 (84%)		
Black	21 (15%)	19 (21%)	2 (4%)		
Other	11 (8%)	5 (5%)	6 (12%)		
Ethnicity				0.57	0.80
Hispanic or Latino	36 (26%)	22 (24%)	14 (29%)		
Not Hispanic or Latino	104 (74%)	69 (76%)	35 (71%)		
Education				0.37	0.65
< High school					
Some college	45 (32%)	31 (34%)	14 (29%)		
College degree	66 (47%)	39 (43%)	27 (55%)		
Graduate degree	29 (21%)	21 (23%)	8 (16%)		
Anthropometric measures					
Weight (kg)	96 ± 16	96 ± 15	95 ± 18	0.83	0.90
BMI (kg/m^2^)	34 ± 4	34 ± 4	34 ± 4	0.97	0.97
Waist circumference (cm)	107 ± 11	107 ± 10	108 ± 12	0.76	0.86
Body fat (%)	41 ± 6	40 ± 6	41 ± 6	0.26	0.60
Cardiorespiratory fitness (mL/kg/min)	25 ± 5	25 ± 5	25 ± 5	0.89	0.91

*Note*: Results (displayed as mean ± SD or *n* [%]); Overall *p* values reflect baseline differences by class membership, analyzed using two sample *t*‐tests for continuous variables or Chi‐Square tests for categorical variables; Statistically significant *p* values (*p* < 0.05) are indicated in bold.

Abbreviations: BMI, body mass index; FDR, false discovery rate.

^a^ Analyzed using Fisher's Exact test using a 3 × 2 table. To compare the proportion of Black participants between the two trajectory groups, Fisher's Exact test *p* value for the 2 × 2 table (Black vs. non‐Black) is < 0.01.

**TABLE 2:** Baseline behavioral and psychosocial predictors of trajectory membership

Baseline predictors	Total sample (*n* = 140)	Weight regainers (*n* = 91)	Weight loss maintainers (*n* = 49)	*p* value	FDR_adj_ *p* value
Behavioral factors					
Bout MVPA (min/d)^a^	25 ± 26	26 ± 28	23 ± 22	0.39	0.65
Energy intake (kcal/d)^b^	1798 ± 487	1817 ± 506	1757 ± 448	0.54	0.80
Fat intake (g/d)^b^	73 ± 25	75 ± 25	70 ± 25	0.30	0.65
Carbohydrate intake (g/d)^b^	206 ± 67	208 ± 69	201 ± 65	0.56	0.80
Protein intake (g/d)^b^	79 ± 22	78 ± 23	80 ± 20	0.68	0.85
Psychosocial factors					
Exercise self‐efficacy	7.14 ± 1.98	7.38 ± 1.99	6.70 ± 1.91	0.05	0.25
Barriers for exercise	29.07 ± 5.70	28.11 ± 5.64	30.8 ± 5.45	**0.01**	0.11
Benefits for exercise^c^	95.89 ± 10.47	96.67 ± 9.93	94.45 ± 11.36	0.25	0.60
External regulation^d^	0.83 ± 0.88	0.77 ± 0.82	0.93 ± 0.97	0.34	0.65
Introjected regulation^d^	1.84 ± 1.04	1.75 ± 1.05	2.01 ± 1.02	0.16	0.51
Identified regulation^d^	2.66 ± 0.77	2.71 ± 0.78	2.58 ± 0.75	0.32	0.65
Intrinsic regulation^d^	2.39 ± 0.99	2.59 ± 0.96	2.04 ± 0.95	**<0.01**	0.06
Eating self efficacy^d^	130.73 ± 28.53	134.6 ± 29.48	123.61 ± 25.49	**0.02**	0.17
Cognitive restraint^d^	8.69 ± 4.12	9.08 ± 4.31	7.98 ± 3.67	0.12	0.44
Disinhibition^e^	8.15 ± 3.30	7.79 ± 3.41	8.81 ± 3.00	0.07	0.31
Hunger^d^	5.96 ± 3.31	5.49 ± 3.26	6.82 ± 3.25	**0.02**	0.17
Weight management behaviors^d^	1.20 ± 0.53	1.25 ± 0.57	1.11 ± 0.46	0.13	0.44
Autonomous regulation^d^	6.20 ± 0.65	6.21 ± 0.68	6.18 ± 0.61	0.76	0.86
Controlled regulation^d^	3.01 ± 1.23	2.91 ± 1.16	3.20 ± 1.33	0.21	0.60
Depressive symptoms^d^ ^,^ ^f^	6.07 ± 6.52	5.27 ± 4.40	7.55 ± 7.18	**0.046**	0.25

*Note*: Results (displayed as mean ± SD or *n* [%]); Overall *p* values reflect baseline differences by class membership, analyzed using two sample *t*‐tests; Statistically significant *p* values (*p* < 0.05) are indicated in bold

Abbreviations: Bout MVPA: minutes of moderate‐to‐vigorous physical activity performed in bouts ≥10 min in duration; FDR: false discovery rate.

^a^ *n* = 133 for total sample; *n* = 86 for weight regainers; *n* = 47 for weight loss maintainers.

^b^ *n* = 136 for total sample; *n* = 87 for weight regainers; *n* = 49 for weight loss maintainers.

^c^ *n* = 138 for total sample; *n* = 89 for weight regainers; *n* = 49 for weight loss maintainers.

^d^ *n* = 139 for total sample; *n* = 90 for weight regainers; *n* = 49 for weight loss maintainers.

^e^ *n* = 137 for total sample; *n* = 89 for weight regainers; *n* = 48 for weight loss maintainers.

^f^ *n* = 5 maintainers and *n* = 2 regainers had depressive symptom scores ≥16.

### 3.3 Association between changes in factors over time with trajectory group

**FIGURE 3:**
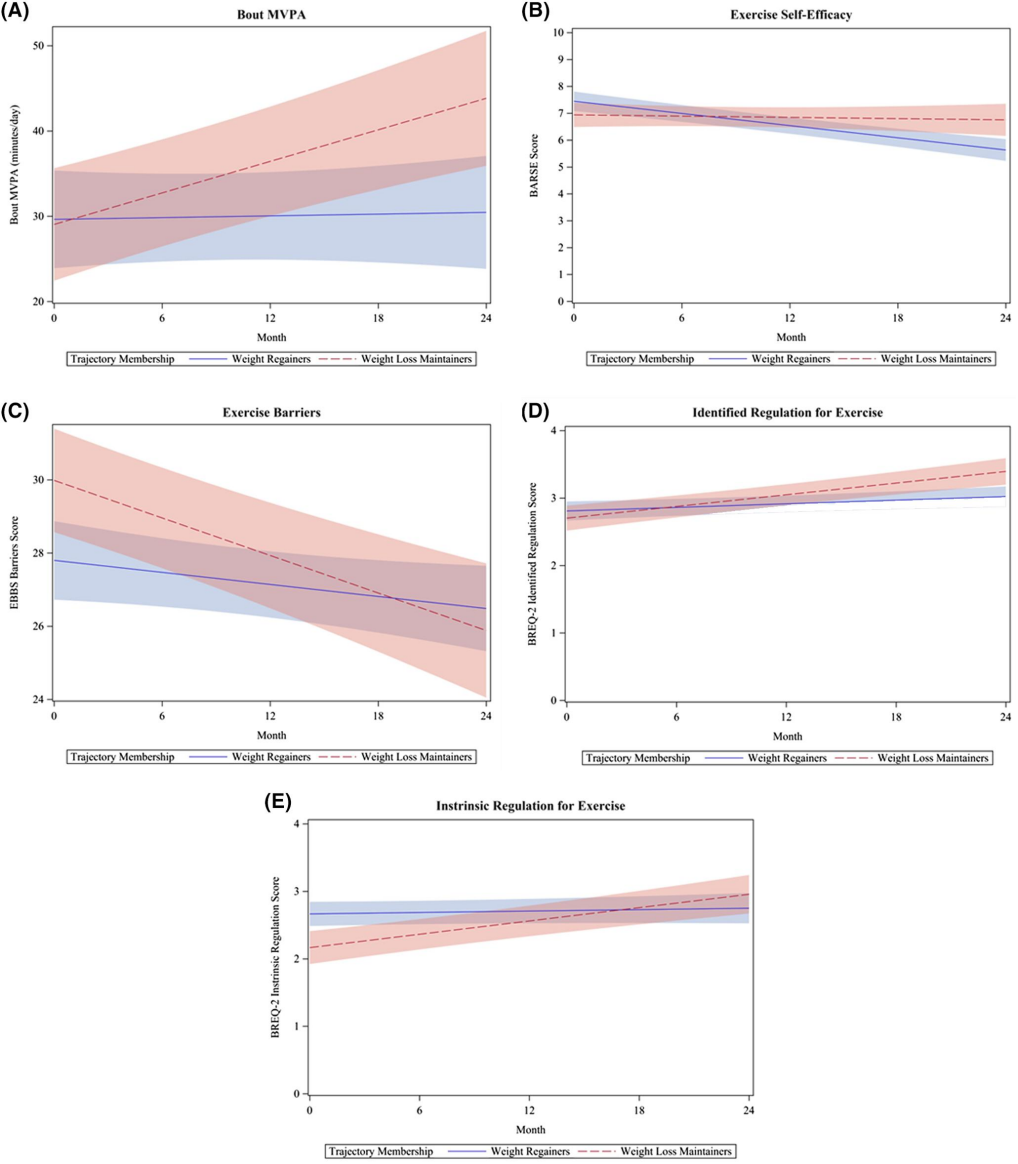
(A–E) Association between changes in exercise‐related behavioral and psychosocial factors over time and trajectory Group. Results from generalized estimating equation models. Panel A displays a significant difference between trajectory groups for change in bout MVPA over time. For every 1 month increase in the study, among weight regainers, bout MVPA increased by 1.9 min/day (95% CI: −1.6, 5.5), while weight loss maintainers increased by 12.1 min/day (95% CI: 7.7, 16.4), on average (false discovery rate [FDR]‐adjusted *p* < 0.01). Panel B displays a significant difference between trajectory groups for change in exercise self‐efficacy (measured with the Barriers Self‐Efficacy Scale) over time. For every 1 month increase in the study, among weight regainers, exercise self‐efficacy decreased by 0.08 units (95% CI: −0.09, −0.06), while weight loss maintainers decreased by 0.008 units (95% CI: −0.04, 0.02) on average (FDR‐adjusted *p* = 0.01). Panel C displays a significant difference between trajectory groups for change in exercise barriers (measured with the Exercise Benefits and Barriers Scale) over time. Weight loss maintainers had, on average, 2.19 (95% CI: 0.41, 3.96) higher score for exercise barriers than weight regainers (FDR‐adjusted *p* = 0.03) at baseline. For every 1 month increase in the study, among weight regainers, exercise barriers decreased by 0.06 units (95% CI: −0.011, 0.0003), while weight loss maintainers decreased by 0.17 units (95% CI: −0.24, −0.11) on average (FDR‐adjusted *p* = 0.01). Panel D displays a significant difference between trajectory groups for change in identified regulation for exercise over time (measured with the Behavioral Regulations for Exercise Questionnaire). For every 1 month increase in the study, among weight regainers, identified regulation for exercise increased by 0.01 units (95% CI: 0, 0.02), while weight loss maintainers increased by 0.03 units (95% CI: 0.02, 0.04) on average (FDR‐adjusted *p* < 0.01). Panel E displays a significant difference between trajectory groups for change in intrinsic regulation for exercise over time (measured with the Behavioral Regulations for Exercise Questionnaire). Weight loss maintainers had, on average, 0.50 (95% CI: 0.20, 0.80) lower score for intrinsic regulation for exercise than weight regainers (FDR‐adjusted *p* < 0.01) at baseline. For every 1 month increase in the study, among weight regainers, intrinsic motivation for exercise increased by 0.004 units (95% CI: 0, 0.01), while weight loss maintainers increased by 0.03 units (95% CI: 0.02, 0.04) on average (FDR‐adjusted *p* < 0.01). Abbreviations: Bout MVPA, minutes of moderate‐to‐vigorous physical activity performed in bouts ≥10 min in duration

There was no significant difference between maintainers and regainers in change in cardiorespiratory fitness (Liters/min; Table S2). Maintainers demonstrated a significant increase in bout MVPA over 24 months, whereas regainers demonstrated a negligible increase (Figure 3A). Maintainers also demonstrated greater adherence to the 300 min/week PA prescription over time compared to weight regainers (*p* = 0.02, FDR adjusted *p* = 0.03, Table S3). There was no significant difference between trajectory groups in changes in average energy intake, macronutrient intake (fat, carbohydrates, protein, Table S2), or adherence to the dietary prescription (Table S3).

Maintainers demonstrated significantly different changes in exercise‐related psychosocial factors over time as compared to regainers, including slower decreases in self‐efficacy for exercise (i.e., both groups demonstrated decreases in self‐efficacy for exercise over time; Figure 3B), greater decreases in barriers for exercise (Figure 3C), and greater increases in identified (Figure 3D), and intrinsic (Figure 3E) regulations for exercise. There was no association between change in perceived benefits for exercise, or external or introjected regulation for exercise and trajectory group (Table S2).

**FIGURE 4:**
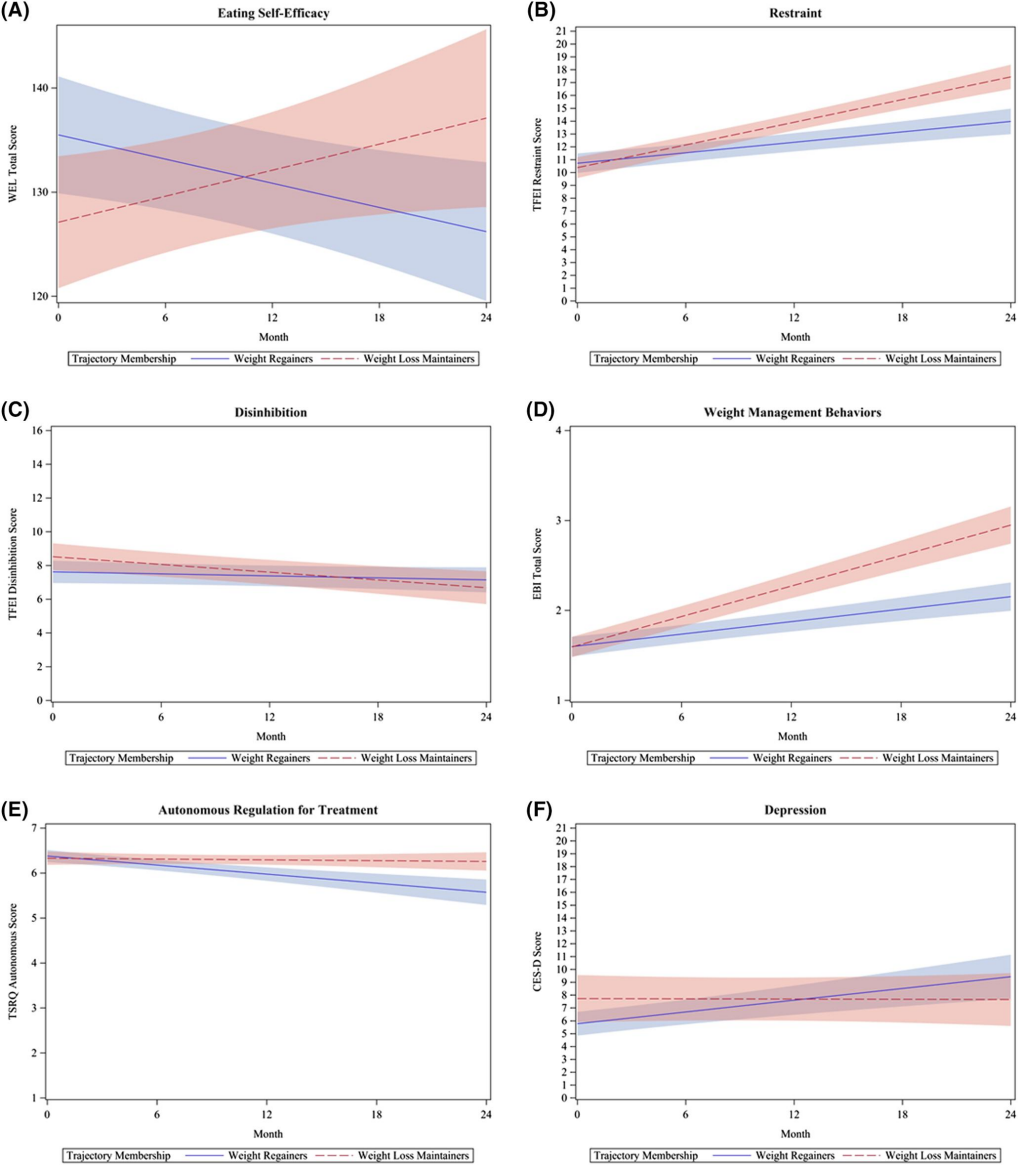
(A–F) Association between change in psychosocial predictors over time and trajectory group. Results from generalized estimating equation models. Panel A displays a significant difference between trajectory groups for change in eating self‐efficacy (measured with the Weight Exercise Lifestyle questionnaire) over time. For every 1 month increase in the study, among weight regainers, eating self‐efficacy decreased by 0.39 units (95% CI: 0.07, 0.70), while weight loss maintainers increased by 0.42 units (95% CI: −0.002, 0.84), on average (false discovery rate [FDR]‐adjusted *p* < 0.01). Panel B displays a significant difference between trajectory groups for change in cognitive restraint (measured with the Three‐Factor Eating Inventory [TFEI]) over time. For every 1 month increase in the study, among weight regainers, cognitive restraint increased by 0.14 units (95% CI: 0.09, 0.18), while weight loss maintainers increased by 0.29 units (95% CI: 0.24, 0.34) on average (FDR‐adjusted *p* < 0.01). Panel C displays a significant difference between trajectory groups for change in disinhibition (measured with TFEI) over time. For every 1 month increase in the study, among weight regainers, disinhibition decreased by 0.02 units (95% CI: −0.05, 0.01), while weight loss maintainers decreased by 0.08 units (95% CI: −0.12, −0.03) on average (FDR‐adjusted *p* = 0.047). Panel D displays a significant difference between trajectory groups for change in self‐reported weight management behaviors (measured using the total score from the Eating Behavior Inventory Revised) over time. For every 1 month increase in the study, among weight regainers, engagement in weight management behaviors increased by 0.02 units (95% CI: 0.02, 0.03), while weight loss maintainers increased by 0.06 units (95% CI: 0.05, 0.06), on average (FDR‐adjusted *p* < 0.01). Panel E displays a significant difference between trajectory groups for change in autonomous motivation to engage in weight loss treatment (measured with the Treatment Self‐Regulation Questionnaire) over time. For every 1 month increase in the study, among weight regainers, autonomous motivation for weight loss treatment decreased by 0.04 units (95% CI: 0.02, 0.04), while weight loss maintainers decreased by 0.003 units (95% CI: −0.01, 0.009) on average (FDR‐adjusted *p* < 0.01). Panel F displays a significant difference between trajectory groups for change in depressive symptoms (measured with the Center for Epidemiologic Studies Depression Scale) over time. For every 1 month increase in the study, among weight regainers, depressive symptoms increased by 0.15 units (95% CI: 0.09, 0.22), while weight loss maintainers decreased by 0.003 units (95% CI: −0.09, 0.08) on average (FDR‐adjusted *p* = 0.01). At 0, 6, 12, 18, and 24 months, respectively, maintainers (*n* = 5, *n* = 6, *n* = 6, *n* = 6, *n* = 2, respectively), and regainers (*n* = 2, *n* = 7, *n* = 13, *n* = 9, *n* = 5, respectively) had depression scores ≥16

Maintainers demonstrated significantly different changes in diet‐related psychosocial factors, including increased eating self‐efficacy (Figure 4A) and cognitive restraint (Figure 4B), decreased disinhibition (Figure 4C), increases in engagement in weight management behaviors (Figure 4D), a slower decrease in autonomous motives for treatment (i.e., both groups demonstrated decreases in autonomous motivation for treatment; Figure 4E), and decreases in depressive symptoms (Figure 4F) over 24 months. Regainers, in contrast, demonstrated decreased eating self‐efficacy, modestly increased restraint, slight decreases in disinhibition, slight increases in engagement in weight management behaviors, decreases in autonomous motives for treatment, and slight increases in depressive symptoms over 24 months. There was no significant between‐group difference in change in hunger or controlled motivation for weight loss treatment (Table S2).

## 4 DISCUSSION

Using a novel data‐driven approach, two distinct weight change trajectory groups were identified: (1) weight regainers, characterized by modest initial weight loss and a regain of initial weight loss by 24 months and (2) weight loss maintainers, characterized by significant initial weight loss and a maintenance of weight loss at 24 months. Black participants were more likely to be classified into the regainer group. Regainers also demonstrated lower baseline barriers to exercise, hunger, and depressive symptoms and higher intrinsic motivation for exercise and eating self‐efficacy as compared to maintainers. Over 24 months, maintainers demonstrated greater increases in MVPA, autonomous motivation for exercise, eating self‐efficacy, cognitive restraint, and engagement in weight management behaviors, and greater reductions in perceived barriers to exercise, disinhibition, and depressive symptoms as compared to regainers. Results from this exploratory analysis provide preliminary evidence that baseline characteristics, and how individuals respond psychologically and behaviorally to an intervention, can predict treatment response. While certain individuals were able to lose and maintain weight with the current intervention, many others were not, and likely need different approaches. Continued research on a wider range of intervention approaches that change individuals' psychology and behavior with regard to eating and PA as well as factors that predict change or weight loss trajectory can lead to a more personalized or precision approach to weight loss and management.

Baseline age, gender, ethnicity, education, body composition, cardiorespiratory fitness, bout MVPA, and energy intake were not predictive of trajectory group. Previous studies have found that age^6^, ^9^ and initial BMI^6^, ^7^, ^9^ at intervention enrollment were important predictors of weight loss maintenance trajectories; however, these findings were not replicated in this analysis, likely due to differences in sample demographics. Our sample was younger (mean ± SD; 40 ± 9 years) compared to the sample in Batterham et al.^6^ (47 ± 9 years), and initial BMI was higher (34 ± 4 kg/m^2^) compared to Batterham et al.^6^ (31 ± 3 kg/m^2^), but lower compared to Morales et al. (37 ± 6 kg/m^2^)^7^ and Courcoulas et al. (median >40 kg/m^2^).^9^ In the present analysis, race was predictive of trajectory group, with 90% of Black participants classified as regainers. However, only 21 Black participants were included in this analytical sample, and after adjustment for multiple comparisons, race was no longer predictive of trajectory group. Thus, these results should be interpreted with caution. In Courcoulas et al.,^9^ race was also predictive of trajectory group such that Black participants were more likely to be classified in the less favorable trajectory groups. Previous research has shown that Black participants, predominantly women, tend to lose less weight than their White counterparts in behavioral weight loss interventions, particularly during the initial 6 months.^28^, ^29^ In the Look AHEAD trial, weight loss at 1 year was significantly lower in Black participants compared to non‐Hispanic Whites (mean ± SE; −6.8 ± 0.3% vs. −9.6 ± 0.2%); however, there were no racial/ethnic differences by Year 4, with the majority of both Black and non‐Hispanic White participants demonstrating significant weight regain.^30^ This racial difference in short‐term weight loss is especially troubling considering that Black adults have higher rates of obesity and obesity‐related comorbidities such as diabetes and cardiovascular disease,^31^ and even short‐term weight loss can have beneficial effects on cardiometabolic risk.^32^ These racial differences in response to weight loss treatment may be a result from a combination of biological, behavioral, and sociocultural factors, which may need to be addressed to optimize weight loss success for Black participants.

One approach to improve treatment response in Black participants may be to consider the racial differences in biological responses to weight loss. For example, in a weight loss trial,^33^ total energy requirements of Black participants were lower, compared to their weight‐matched White counterparts. These biological differences could have major implications for caloric intake prescriptions in a weight loss intervention, as Black participants may need lower weight loss calorie goals^33^ and perhaps more tailored behavioral support to achieve lower calorie goals. Because Kinsey et al. found the Black participants achieved similar weight loss to their White counterparts when Black participants made up >50% of the sample, another approach may be to increase the overall proportion of Black participants, which may naturally alter group dynamics and interpersonal relationships.^34^ Alternatively, other studies have culturally adapted the behavioral intervention by tailoring print materials to be more representative of minority participants, providing culturally based food and activity recommendations, and/or inclusion of spirituality in intervention messaging. Some studies show that culturally adapting interventions results in enhanced weight loss and weight loss maintenance in Black participants,^2^, ^35^, ^36^ while others show that cultural adaptations did not have an additional impact on weight loss or maintenance outcomes in behavioral interventions.^37^ Although the majority of Black participants in the present sample were classified to the regainer group, 68% of White, and 61% of Hispanic or Latino participants were also classified as weight regainers, indicating that long‐term weight loss maintenance remains critical challenge in obesity treatment for all races/ethnicities. Future studies should continue to explore specific strategies that improve weight loss and weight loss maintenance amongst all participants.

Several psychosocial factors at baseline were predictive of trajectory group, but in the opposite expected directions. Surprisingly, regainers started the intervention with lower perceived barriers for exercise, higher intrinsic motivation for exercise (enjoyment), higher eating self‐efficacy, lower hunger, and lower depressive symptoms as compared to maintainers. It is possible that regainers over‐estimated their eating self‐efficacy for various eating occasions (e.g., when anxious/nervous or while watching TV). Work by McAuley et al. suggests that, at baseline, participants may not have the appropriate previous experiences to form accurate efficacy expectations, and thus they over‐estimate their capabilities at baseline.^38^ Alternatively, these results may suggest a ceiling effect, considering the regainers did not have much room to improve and may have been less likely to benefit from a behavioral weight loss intervention. If replicated in larger samples, these approaches may be used to identify individuals who may need alternative behavioral support or earlier initiation of adjunctive strategies such as pharmacotherapy or bariatric surgery to be successful with a behavioral weight loss intervention.

Maintainers demonstrated significantly greater increases in bout MVPA over 24 months as compared to regainers. High levels of PA (250–300 min/week) are a critical component of successful weight loss maintenance and are universally recommended to prevent weight regain.^39^ In the present study, maintainers accumulated approximately 300 min/week of bout MVPA at 24 months, which is well aligned with current PA guidelines for weight management. In contrast, regainers accumulated approximately 215 min/week of bout MVPA at 24 months. Though it is possible to maintain weight loss without high volumes of PA,^40^ results from the present study support previous findings indicating that high levels of PA may be critical for long‐term weight loss maintenance.^41^ A previous study found that factors related to energy intake, such as reductions in perceived barriers to healthy eating and lower fat intake, were associated with favorable weight loss maintenance trajectories.^5^ However, no significant relationship between weight trajectory group and changes in dietary energy intake or macronutrient composition was observed. It is well‐established that self‐reported measures of dietary intake are prone to error and misreporting^42^ and thus, our failure to find relationships between weight loss and these variable should be interpreted with caution.

Motivation for exercise may be one factor that impacts PA adherence and in turn, weight loss maintenance success. At baseline, maintainers had lower intrinsic regulation compared to regainers; however, at 24 months, maintainers demonstrated an increase in autonomous motivation for exercise (identified and intrinsic regulations). Previous literature examining the role of motivation for exercise has identified a beneficial role of autonomous motives in predicting maintenance of PA.^43^ Our results support this idea and demonstrate that increases in motivation to engage in exercise for health benefits or the pure enjoyment of activity are related to long‐term weight loss maintenance success. Another factor to consider in maximizing weight loss maintenance is reducing barriers to exercise. Time, energy levels, resources (distance to places to be physically active, costs, fitness center schedules), and social aspects (feelings of embarrassment, lack of social support) represent common reported barriers to increasing PA.^44^ In the present study, all participants were provided a complimentary fitness center membership and exercise behavioral support. It remains unclear why regainers did not respond well to the PA intervention. Future interventions may improve PA adherence, and thus long‐term weight loss, by promoting autonomy and activities that participants find personally meaningful and enjoyable. For example, to promote autonomy and PA enjoyment, PA interventions could allow participants to choose their PA type, location (more aesthetically pleasing settings such as outdoors), intensity, and duration.^45^ However, future studies are needed to determine whether these approaches promote long‐term weight loss.

Significant improvements in eating self‐efficacy, cognitive restraint, disinhibition, and engagement in weight management behaviors were observed in maintainers as compared to regainers, which are consistent with previous findings. Eating self‐efficacy is an important predictor of the adoption and maintenance of weight control behaviors including restricting caloric intake.^46^ In addition, increases in cognitive restraint and reductions in disinhibition during weight loss interventions are associated with long‐term weight loss and maintenance.^47^ Thus, improving eating behaviors may be critical for success (e.g., incorporate individualized support from a registered dietitian, portion control strategies, and/or planning skills, among others).^44^

There are several important limitations to note. First, this was a secondary analysis, and the original weight loss trial was not powered to perform cluster analyses. As a result, the sample size was limited and contributed to the substantial individual variability observed within the two trajectory groups. Future studies with larger sample sizes should apply this approach to improve our understanding of this underlying variability in response to weight loss treatment. Despite a small sample size, the majority of participants (79%) provided ≥6 weights over 24 months, representing a study strength. Second, like many other weight loss interventions, the majority of participants were White women. However, distribution of racial (15% Black) and Hispanic or Latino (26%) minorities exceeded or was similar to that of Denver County (10% Black and 29% Hispanic or Latino).^48^ An important study strength is the consideration of several biological, behavioral, and psychosocial factors recommended by the Accumulating Data to Optimally Predict Obesity Treatment (ADOPT), project.^4^ Future studies should include planned assessments and analyses of additional biological (e.g., resting energy expenditure, hormones related to appetite and hunger), behavioral (sleep), and psychosocial variables (e.g., stress and personality) related to weight loss not included in this study as well as environmental variables (e.g., perceptions of crime, walkability, food environment) that may contribute to changes in weight over time. Ultimately, these approaches will improve our understanding of the determinants of different weight loss trajectories in response to interventions in adults with overweight and obesity.

Results from this exploratory study identified two distinct weight loss trajectories over 24 months that exhibited significantly different patterns of weight change over time. This is one of the first studies to comprehensively evaluate sociodemographic, as well as baseline levels and changes over time in biologic, behavioral, and psychosocial factors associated with trajectory groups. At baseline, regainers demonstrated higher intrinsic motivation for exercise, eating self‐efficacy, hunger, and lower barriers for exercise and depressive symptoms, suggesting that this group may not have had as much room to improve and thus may have been less likely to benefit from a behavioral weight loss intervention, or that they may require alternative behavioral intervention approaches. Notably, the majority of Black participants were in the regainer group. Future weight loss interventions should consider the unique needs of Black participants to promote health equity and greater treatment efficacy. Maintainers demonstrated greater improvements in bout MVPA, autonomous motivation for exercise, eating self‐efficacy, cognitive restraint, disinhibition, weight management behaviors, and depressive symptoms over 24 months as compared to regainers. Our findings extend the existing literature and provide a comprehensive exploration of several factors that may contribute to the underlying reasons for different responses to weight loss treatment. This analysis represents an alternative, data‐driven approach to probe candidate factors that may predict success or failure in weight loss interventions, and suggest potential targets for more tailored, multifaceted interventions to maximize treatment success. Future studies should validate these findings in other prospective weight loss interventions.

## CONFLICT OF INTEREST

Nia S. Mitchell has disclosed a grant from the National Institutes of Health, unrelated to the current work and Suzanne Phelan has disclosed a grant from WW, International, unrelated to the current work. All other coauthors have nothing to disclose.

## AUTHOR CONTRIBUTIONS

**Victoria A. Catenacci, Daniel H. Bessesen**, and **Edward L. Melanson** conceived of and designed the study and obtained funding. Victoria A. Catenacci wrote the protocol and acquired the data. Laura Grau and Jaron Arbet performed the statistical analysis and created data visualizations. All authors assisted with interpretation of the data. Danielle M. Ostendorf and Jennifer M. Blankenship drafted the manuscript. Danielle M. Ostendorf and Laura Grau generated tables and figures. All authors were involved in writing and revising the manuscript and approved the final version of the manuscript.

## Supporting information

Supporting Information 1
