# Supplementary material for: Predictors of long‐term weight loss trajectories during a behavioral weight loss intervention: An exploratory analysis
Source: Obes Sci Pract. 2021 May 19;7(5):569–82. doi: 10.1002/osp4.530 (PMC8488452; doi:10.1002/osp4.530)
Supplement: Supplementary file 1 — Supporting Information 1 [file OSP4-7-569-s001.docx]

**Supporting Information**

**TITLE:** Predictors of Long-term Weight Loss Trajectories during a Behavioral Weight Loss Intervention

**AUTHORS:** Danielle M. Ostendorf * ^1, 2^, Jennifer M. Blankenship * ^2^, Laura Grau ^3^, Jaron Arbet ^3^, Nia S. Mitchell ^4^, Seth A. Creasy ^1,2^, Ann E. Caldwell ^1,2^, Edward L. Melanson ^2, 5-6^, Suzanne Phelan ^7^, Daniel H Bessesen ^1,2^, and Victoria A. Catenacci ^1, 2^

**AFFILIATIONS:**

* These authors contributed equally to this work

^1^ Department of Medicine, Anschutz Health and Wellness Center, University of Colorado Anschutz Medical Campus, Aurora, CO, USA

^2^ Department of Medicine, Division of Endocrinology, Metabolism, and Diabetes, University of Colorado Anschutz Medical Campus, Aurora, CO, USA

^3^ Department of Biostatistics and Informatics, Colorado School of Public Health, University of Colorado Anschutz Medical Campus, Aurora, CO, USA

^4^ Department of Medicine, Duke University School of Medicine, Durham, NC, USA

^5^ Department of Medicine, Division of Geriatric Medicine, University of Colorado Anschutz Medical Campus, Aurora, CO, USA

^6^ Eastern Colorado Veterans Affairs Geriatric Research, Education, and Clinical Center, Denver, CO, USA

^7^ Department of Kinesiology & Public Health, California Polytechnic State University, San Luis Obispo, CA, USA

**CONTACT INFO:**

Danielle M. Ostendorf, PhD

University of Colorado Anschutz Medical Campus, Campus Box C263

12348 E. Montview Boulevard, Aurora, CO  80045

[Danielle.ostendorf@cuanschutz.edu](mailto:Danielle.ostendorf@cuanschutz.edu)

**Supplementary Table S1: Comparison of Latent Class Growth Mixture Models and Model Selection**

| **Clusters** | **Trajectory**  **Shape** | **Random**  **Effects** | **npm** | **BIC** | **N(%) class 1** | **N(%) class 2** | **N(%) class 3** |
| --- | --- | --- | --- | --- | --- | --- | --- |
| **2** | **Cubic** | **Yes** | **21** | **4222.9** | **49(0.35)** | **91(0.65)** |  |
| 1 | Cubic | Yes | 15 | 4228.5 | 140(1) |  |  |
| 1 | Quartic | Yes | 21 | 4232.7 | 140(1) |  |  |
| 2 | Quartic | Yes | 28 | 4232.9 | 47(0.34) | 93(0.66) |  |
| 3 | Cubic | Yes | 27 | 4241.2 | 42(0.3) | 87(0.62) | 11(0.08) |
| 3 | Quartic | Yes | 35 | 4254.7 | 35(0.25) | 94(0.67) | 11(0.08) |
| 2 | Quadratic | Yes | 15 | 4395.8 | 55(0.39) | 85(0.61) |  |
| 1 | Quadratic | Yes | 10 | 4402.1 | 140(1) |  |  |
| 3 | Quadratic | Yes | 20 | 4415.5 | 68(0.49) | 44(0.31) | 28(0.2) |
| 2 | Linear | Yes | 10 | 4747.9 | 51(0.36) | 89(0.64) |  |
| 1 | Linear | Yes | 6 | 4760.4 | 140(1) |  |  |
| 3 | Linear | Yes | 14 | 4764 | 58(0.41) | 41(0.29) | 41(0.29) |
| 3 | Quadratic | No | 12 | 4956.2 | 24(0.17) | 75(0.54) | 41(0.29) |
| 3 | Cubic | No | 15 | 4958.5 | 24(0.17) | 42(0.3) | 74(0.53) |
| 3 | Quartic | No | 18 | 4972.5 | 24(0.17) | 42(0.3) | 74(0.53) |
| 3 | Linear | No | 9 | 5054.1 | 23(0.16) | 42(0.3) | 75(0.54) |
| 2 | Quadratic | No | 8 | 5177.2 | 39(0.28) | 101(0.72) |  |
| 2 | Cubic | No | 10 | 5181.3 | 38(0.27) | 102(0.73) |  |
| 2 | Quartic | No | 12 | 5190.8 | 38(0.27) | 102(0.73) |  |
| 2 | Linear | No | 6 | 5236.9 | 39(0.28) | 101(0.72) |  |
| 1 | Quadratic | No | 4 | 5763.2 | 140(1) |  |  |
| 1 | Cubic | No | 5 | 5766.2 | 140(1) |  |  |
| 1 | Quartic | No | 6 | 5771.1 | 140(1) |  |  |
| 1 | Linear | No | 3 | 5776.2 | 140(1) |  |  |

*Note.* Table is sorted from best to worst by Bayesian Information Criteria (BIC); npm: number of estimated parameters; Random effects: yes indicates that random subject-specific effects were included (subject-specific baseline and change over time) where the random effects covariance matrix was allowed to differ within each cluster, and no indicates that no random effects were included

**Supplementary Table S2: Association between Change in Biologic, Behavioral, and Psychosocial Factors over Time and Trajectory Group**

| **Predictor** | **Parameter** | **Estimate** | **95% CI** | **Raw *P* Value** | **FDR_adj_ *P* Value** |
| --- | --- | --- | --- | --- | --- |
| ***Biologic Factors*** | | | | | |
| Cardiorespiratory Fitness (Liters/min) | Intercept | 2.3776 | (2.25,2.5) | **<.01** | **<.01** |
|  | Trajectory | -0.0239 | (-0.21,0.16) | 0.80 | 0.83 |
|  | Month | 0.0019 | (0,0) | 0.13 | 0.17 |
| ***Behavioral Factors*** | | | | | |
| Energy Intake (kcal/day) | Intercept | 1769.78 | (1695.52,1844.04) | **<.01** | **<.01** |
|  | Trajectory | -26.1182 | (-101.59,49.35) | 0.50 | 0.58 |
|  | Month | -17.9002 | (-24.01,-11.79) | **<.01** | **<.01** |
| Total Fat (average grams/day) | Intercept | 73.1772 | (69.38,76.97) | **<.01** | **<.01** |
|  | Trajectory | -1.9916 | (-6.45,2.47) | 0.38 | 0.47 |
|  | Month | -0.892 | (-1.19,-0.6) | **<.01** | **<.01** |
| Total Carbohydrates (average grams/day) | Intercept | 211.807 | (201.31,222.3) | **<.01** | **<.01** |
|  | Trajectory | -3.1794 | (-13.35,6.99) | 0.54 | 0.62 |
|  | Month | -2.384 | (-3.2,-1.57) | **<.01** | **<.01** |
| Total Protein (average grams/day) | Intercept | 78.5676 | (74.82,82.31) | **<.01** | **<.01** |
|  | Trajectory | 0.9113 | (-3.48,5.31) | 0.68 | 0.73 |
|  | Month | -0.4999 | (-0.79,-0.21) | **<.01** | **<.01** |
| ***Psychosocial Factors*** | | | | | |
| Benefits for Exercise | Intercept | 96.4393 | (94.5,98.38) | **<.01** | **<.01** |
|  | Trajectory | -0.4968 | (-3.58,2.59) | 0.75 | 0.79 |
|  | Month | 0.0275 | (-0.05,0.1) | 0.48 | 0.57 |
| External Motivation for Exercise | Intercept | 0.825 | (0.67,0.98) | **<.01** | **<.01** |
|  | Trajectory | 0.0463 | (-0.2,0.29) | 0.71 | 0.76 |
|  | Month | -0.0091 | (-0.01,0) | **<.01** | **<.01** |
| Introjected Motivation for Exercise | Intercept | 1.8391 | (1.65,2.03) | **<.01** | **<.01** |
|  | Trajectory | 0.1607 | (-0.12,0.45) | 0.27 | 0.36 |
|  | Month | 0.007 | (0,0.01) | 0.08 | 0.11 |
| Hunger | Intercept | 5.4085 | (4.81,6.01) | **<.01** | **<.01** |
|  | Trajectory | 0.6438 | (-0.29,1.58) | 0.18 | 0.24 |
|  | Month | -0.0612 | (-0.09,-0.04) | **<.01** | **<.01** |
| Controlled Motivation for Treatment | Intercept | 3.1432 | (2.92,3.36) | **<.01** | **<.01** |
|  | Trajectory | 0.1275 | (-0.21,0.47) | 0.46 | 0.54 |
|  | Month | -0.0084 | (-0.02,0) | 0.08 | 0.11 |

*Note.* Results from generalized estimating equation models with continuous outcomes. The Trajectory term compares weight loss maintainers vs. regainers (reference group = regainers). An interaction between Month and Trajectory was only included if the interaction *P* value was < 0.05. Statistically significant *P* values (*P*<0.05) are indicated in bold; FDR: false discovery rate.

**Supplementary Table S3: Association between Change in Adherence to Diet and Physical Activity Prescriptions over Time and Trajectory Group**

| **Predictor** | **Parameter** | **Odds Ratio** | **95% CI** | **Raw *P* Value** | **FDR_adj_ *P* Value** |
| --- | --- | --- | --- | --- | --- |
| Dietary Adherence | Intercept | 0.3308 | (0.23,0.48) | **<.01** | **<.01** |
|  | Trajectory | 0.8267 | (0.53,1.29) | 0.40 | 0.49 |
|  | Month | 1.08351 | (1.05,1.12) | **<.01** | **<.01** |
| Physical Activity Adherence | Intercept | 0.3636 | (0.24,0.55) | **<.01** | **<.01** |
|  | Trajectory | 1.09361 | (0.56,2.12) | 0.79 | 0.82 |
|  | Month | 1.09567 | (0.86,1.39) | 0.45 | 0.54 |
|  | Month*Trajectory | 1.67869 | (1.11,2.55) | **0.02** | **0.03** |

*Note.* Results from logistic generalized estimating equation models with a binary longitudinal variable as the outcome. The Trajectory term compares weight loss maintainers vs. regainers (reference group = regainers). An interaction between Month and Trajectory was only included if the interaction *P* value was < 0.05. Statistically significant *P* values (*P*<0.05) are indicated in bold; FDR: false discovery rate. A participant was defined as adherent to the dietary prescription, if their estimated energy intake (kcals/day) was at or below their prescribed calorie goal (average energy intake ≤ energy intake prescription, yes/no), and months including 0, 6, 12, and 18 for both standard and sequential. A participant was defined as adherent to the physical activity prescription if their device-measured bout moderate-to-vigorous physical activity (MVPA) was at or above the prescribed physical activity level (bout MVPA ≥ 300 minutes/week, yes/no) at each time point (months 6, 12, and 18 for standard; months 12, 18, and 24 for sequential).
